# CAR-macrophages targets CD26 to eliminate chronic myeloid leukemia stem cells

**DOI:** 10.1186/s40164-025-00608-9

**Published:** 2025-02-13

**Authors:** Jiang Guoyun, Qin Yuefeng, Huang Zhenglan, Yuan Zuowei, Zhou Hongyan, Yuan Ying, Feng Wenli

**Affiliations:** 1 Department of Clinical Hematology, School of Laboratory Medicine, Chongqing Medical University, No. 1, Yixueyuan Road, Yuzhong District, Chongqing, 400016 China; 2 Department of Respiratory and Critical Care Medicine, The First Affiliated Hospital of Chongqing Medical University, No. 1, Youyi Road, Yuzhong District, Chongqing, 400016 China

**Keywords:** Chronic myeloid leukemia, Chimeric antigen receptor, Macrophage, CD26

## Abstract

**Background:**

Chronic myeloid leukemia stem cells (CML-LSCs), which exhibit resistance to tyrosine kinase inhibitors (TKIs), are the leading cause of treatment failure and recurrence in chronic myeloid leukemia (CML). This highlights the urgent need for novel therapies aimed at eliminating these CML-LSCs. Chimeric antigen receptor macrophages (CAR-M) not only perform phagocytosis on target cells but also function as antigen-presenting cells, thereby activating the anti-tumor immune response.CD26 (dipeptidyl peptidase 4, DPP IV) is abundantly expressed in CML-LSCs and functions as a tumor-specific antigen (TSA) in CAR-M treatment. The purpose of this study is to evaluate CAR-M’s efficacy in targeting CD26-positive CML cells and to develop a novel strategy for CML treatment.

**Methods:**

CD26 CAR-M was constructed using mouse-derived macrophage Raw264.7 cells. CD26 was overexpressed in CML cell lines BP210 and BP210-T315I. The targeting phagocytosis of CAR-M was verified using confocal microscopy and flow cytometry. X-ray was used to eliminate the tumorigenicity of CAR-M, and the safety of CAR-M was verified through CCK-8, clone formation assays, and animal experiments. To assess the anti-leukemia ability of CAR-M in the CML mouse model, the survival, peripheral blood white blood cell counts, and CML cell infiltration in the liver, spleen, and bone marrow (BM) were measured. Additionally, CD26 CAR-THP1 was constructed, and its phagocytic ability against CD26-positive cells NCI-H2452 was confirmed by confocal microscopy.

**Results:**

We successfully constructed CD26 CAR-M and validated its targeted phagocytosis of CD26-positive CML cells both in vitro and in vivo. The data indicate that CAR-M has higher phagocytic efficiency in CD26-positive CML cells than in CD26-negative cells. CAR-M-treated CML mice demonstrated extended survival and reduced CML invasion. In addition, CAR-THP1 demonstrated targeted phagocytosis of NCI-H2452 cells that normally express CD26.

**Conclusion:**

This study demonstrates that CD26 CAR-M effectively targets and phagocytizes CD26-positive CML cells, implying that targeting CD26 with CAR-M could be a viable method for eradicating CML-LSCs. Furthermore, our discoveries illuminate the potential application of CAR-M in treating hematological malignancies.

**Supplementary Information:**

The online version contains supplementary material available at 10.1186/s40164-025-00608-9.

## Introduction

Chronic myeloid leukemia (CML) is a malignant proliferative disorder that affects hematopoietic stem cells in the bone marrow (BM). The hallmark of CML is the presence of the bcr-abl1 fusion gene, which encodes the BCR::ABL1 oncoprotein. This oncoprotein activates several downstream signaling pathways, including RAS/MAPK, PI3K/AKT, and STAT5, thereby promoting cell proliferation, inhibiting apoptosis, and ultimately driving malignant transformation [1, 2]. Tyrosine kinase inhibitors (TKIs) function by competitively inhibiting the tyrosine kinase activity of BCR::ABL1 through binding to its ATP-binding site. Currently, four TKIs are approved for use as the frontline treatment for newly diagnosed chronic phase CML (CML-CP), including imatinib, dasatinib, bosutinib, and nilotinib [3]. However, TKIs are not curative, requiring patients to remain on long-term therapy. Furthermore, patients face challenges such as TKIs resistance, disease relapse, and progression [1]. Studies indicate that 15–25% of CML patients in the chronic phase exhibit resistance to TKIs therapy, and approximately 18% of patients who achieve complete remission experience relapse within five years after discontinuing treatment [4, 5]. The persistence of chronic myeloid leukemia stem cells (CML-LSCs), which are intrinsically resistant to TKIs, is believed to be the primary driver of CML relapse and progression [6]. As a result, in cases of disease recurrence or rapid transformation, treatment outcomes remain suboptimal, highlighting the urgent need for novel therapeutic approaches.

Chimeric antigen receptor (CAR) therapy has emerged as a promising cancer treatment due to its high specificity in antigen recognition and potent tumor cell destruction [7]. CAR is a genetically engineered cell surface receptor that enables immune cells to target specific antigenic proteins. Structurally, the extracellular domain of CAR comprises a single-chain variable fragment (scFv), a hinge region, and a transmembrane domain, while the intracellular domain includes a costimulatory domain and a signal transduction domain [8]. CAR-modified immune cells are generated in vitro and subsequently reinfused into the patient. Upon recognizing tumor-specific antigen (TSA) through the scFv, the immune cells are activated to destroy the tumor cells. While CAR therapy aims to target TSA on the surface of tumor cells, CML cells lack a special membrane antigen, making CAR-based immunotherapy challenging. CD26 (dipeptidyl peptidase 4, DPP IV) is a specific marker of CML-LSCs, with high expression in CML-LSCs and exceedingly low in normal stem cells [9, 10]. After TKIs treatment, primitive and quiescent CML-LSCs continue to express CD26. Except for active lymphocytes, other normal blood cells seldom express CD26, implying that targeting CD26 may represent a viable strategy for eliminating CML-LSCs [11, 12]. Therefore, CD26-targeted CAR therapy holds the potential to address the critical issues of drug resistance and relapse associated with CML-LSCs.

CAR-T, CAR natural killer (CAR-NK), and CAR macrophages (CAR-M) are innovative cell therapies that based on CAR technology and have similar structural components [13]. CAR-T cells are genetically engineered T cells designed to specifically target TSA on cancer cells. CAR-T therapy offers high specificity, potent cytotoxicity, and long-lasting effects, and does not rely on human leukocyte antigen (HLA) for tumor eradication. It has been successfully applied in the treatment of various cancers, with the most notable results observed in CD19-targeted CAR-T therapy for malignant B-cell tumors [14]. However, CAR-T therapy is associated with adverse effects such as cytokine release syndrome (CRS) and neurotoxicity, and the phenomenon of CAR-T cell exhaustion further limits its efficacy [15]. In our previous work, we developed CD26-targeted CAR-T cells. However, since CD26 is also expressed in T cells, CD26 CAR-T cells exert cytotoxic effects on themselves, leading to self-destruction (Fig. S1A-C). In contrast, CAR-NK therapy offers a shorter lifespan, which reduces the risk of CRS and neurotoxicity. Nevertheless, due to the inherent properties of NK cells, they are resistant to lentiviral transduction, making gene modification and the use of lentivirus-based systems for intracellular transduction challenging [16]. We also encountered difficulties with lentiviral infection of NK-92 cells while generating CD26 CAR-NK (Fig. S1D). Macrophages, known for their exceptional phagocytic activity and antigen presentation capabilities, serve as the foundation for CAR-M therapy. CAR-M not only directly kills tumor cells but also secretes pro-inflammatory cytokines and presents tumor antigens to T cells, thereby activating the adaptive immune response [17, 18]. Compared to CAR-T and CAR-NK therapies, CAR-M therapy exhibits reduced non-tumor toxicity due to its shorter circulation time [13]. Additionally, CAR-M is capable of infiltrating the tumor microenvironment, making it a particularly attractive option for solid tumor treatment [19]. However, the potential of CAR-M in targeting hematologic malignancies remains to be thoroughly explored.

In this study, we successfully engineered CD26-targeted CAR-M and demonstrated their efficacy in selectively eliminating CD26-positive CML cells both in vitro and in vivo. This study expands the investigation of CAR-M therapy in hematologic malignancies and suggests that CAR-M therapy could be a promising approach to combat drug resistance and relapse in CML patients.

## Materials and methods

### Cells

The human monocyte line THP1 was purchased from the Cell Culture Center of the Chinese Medical Science Academy in Shanghai. The mouse monocyte line RAW264.7 was donated by Dr. Chen Xi from the First Affiliated Hospital of Chongqing Medical University. The BP210 and BP210-T315I cell lines were created by transfecting BaF3 cells (a murine primary B lymphoblastic cell line) with P210^BCR − ABL^ or P210^BCR − ABL−T315I^ expressing retroviruses [20, 21].THP1, BP210, and BP210-T315I were cultured in RPMI 1640 medium with 10% fetal bovine serum (FBS, Gibco). Raw264.7 cells were cultured in DMEM medium with 10% FBS.

### Construction and identification of CAR-M

CAR-M was constructed using the mouse-derived macrophage line RAW264.7. Briefly, 2 × 10⁴ RAW264.7 cells were seeded into 96-well plates. After cell adhesion, CD26 CAR lentivirus (MOI = 30) and polybrene were added. Wild-type macrophages (WT-M) were constructed by negative control lentivirus. Flow cytometry (FCM) and fluorescence microscopy were employed to assess the percentage of GFP-positive cells, Western blot analysis to detect CD26 CAR protein expression, and quantitative reverse transcription PCR (qRT-PCR) to assess CD26 CAR mRNA levels. The human monocyte cell line THP1 was also infected with the CD26 CAR lentivirus or negative control lentivirus (MOI = 30) to generate CD26 CAR-THP1 or Control THP1 (Con-THP1).

### Construction and verification of CD26-overexpressing CML cells

Mouse CML cell lines BP210 and BP210-T315I were adjusted to 10,000 cells/100 µl, and CD26-overexpressing lentivirus (MOI = 30) with polybrene was added. Stably transfected cells were selected using puromycin. The expression of CD26 was confirmed by FCM, Western blot, and qRT-PCR analysis.

### CAR-M targeting phagocytosis in vitro

CD26⁺ BP210 and CD26⁺ BP210-T315I cells were collected and co-cultured with CAR-M cells. Phagocytosis rates were determined by FCM, where the ratio of fluorescent double-positive cells (red fluorescence for CD26⁺ CML cells and CAR-M cells labeled with APC anti-CD11b antibody [BioLegend, #101211]) was assessed. After 24–48 h of co-culture, the phagocytic ability of CAR-M cells was further evaluated using confocal laser scanning microscopy (CLSM) (red fluorescence for CD26⁺ CML cells and GFP for CAR-M cells). Wright’s staining was used to assess the polarization and phagocytosis effects of CAR-M cells.

### The targeted phagocytosis of CAR-THP1

NCI-H2452 cells were transfected with a lentivirus carrying red fluorescent protein (RFP) at an MOI of 30. CAR-THP1 and Con-THP1 were treated with Phorbol-12-myristate-13-acetate (PMA) for 48 h. Then, CAR-THP1 and Con-THP1 cells were harvested and co-cultured with NCI-H2452 cells at a 5:1 ratio in 12-well plates. After 48 h, the phagocytosis effect of CAR-THP1 cells was assessed using CLSM.

### The differentiation of THP1 to macrophages

Before the phagocytosis experiments, CAR-THP1 cells were treated with 100 ng/ml PMA for 48 h to induce differentiation into macrophages. After PMA treatment, THP1 cell adhesion was examined using microscopy, and CD11b expression was measured by qRT-PCR. FCM detected the proportion of CD11b-positive cells using the APC anti-CD11b antibody (BioLegend, #101211).

### RNA extraction and qRT-PCR

RNA was isolated using AG RNAex Pro RNA reagent (Accurate Biology, #AG21102) and reverse transcribed using Evo M-MLV RT Kit for qPCR (Accurate Biology, #AG11707). The qRT-PCR was carried out through SYBR Green Premix Pro Taq HS qPCR Kit (Accurate Biology, #AG11701) following the product’s instructions. β-Actin was used as qRT-PCR internal parameters. Relative expression was normalized to the geometric mean of housekeeping gene expression and was calculated using the 2-ΔΔCt method. All specific primers sequences used in this investigation were listed in Supplementary Table.

### The safety evaluation of CAR-M cells

Because RAW264.7 cells are tumorigenic, they were irradiated with X-rays before use. The optimal radiation dose was determined before investigating CAR-M function in vivo. CAR-M cells were irradiated with X-rays at doses of 0, 2.5, 5, 7.5, or 10 Gy, and cell proliferation was assessed using CCK-8 and colony-forming assays. For the CCK-8 assay, 3,000 CAR-M cells were seeded in 96-well plates, and at specific time points, 10 µl of CCK-8 reagent (Topscience, China) was added and incubated at 37 °C for 3 h. The optical density (OD) value was measured at 450 nm (BioTek, USA), and the proliferation rate was calculated. For the colony-forming assay, 300 CAR-M cells were seeded in 12-well plates and incubated for 7 days. Cells were then fixed with 4% paraformaldehyde, stained with crystal violet (Beyotime Biotechnology, China), washed with PBS, scanned, and analyzed for colony number using ImageJ software. To assess the in vivo safety of CAR-M, female BALB/c mice (5–6 weeks old) were randomly assigned to three groups (*n* = 6). Mice received subcutaneous injections of 1 × 10⁷ untreated, 5 Gy-irradiated, or 10 Gy-irradiated CAR-M cells. Mouse survival time, body weight, and tumor size were monitored every three days. After 21 days, tumors were weighed, and histological analysis was performed using hematoxylin and eosin (HE) staining.

### CAR-M’s anti-leukemia capability in vivo

Female BALB/c mice aged 5–6 weeks were randomly divided into six groups (*n* = 6). On day 0, mice receive either 1 × 10^7^ CD26^+^ BP210 or CD26^+^ BP210-T315I via the tail vein. On day 7, 1 × 10^7^ irradiated CAR-M cells were injected into the experimental group via the tail vein. The control group got the same quantity of irradiated WT-M cells, while the blank group was given PBS. The treatment was repeated every 7 days for three times. The survival time and peripheral blood white blood cell (WBC) count of mice were monitored, and the infliction of CML cells in the liver, spleen, and BM of mice was detected by Wright’s staining. The expression of BCR::ABL1 oncoprotein was measured by immunofluorescence staining.

### Statistical analysis

Statistical analysis was performed using GraphPad Prism 8.0 software. The data was presented as a mean ± SD. The statistical significance between groups was determined using one-way ANOVA analysis. A p-value < 0.05 was considered statistically significant.

## Results

### Generation and characterization of CD26 CAR-M

**Fig. 1:**
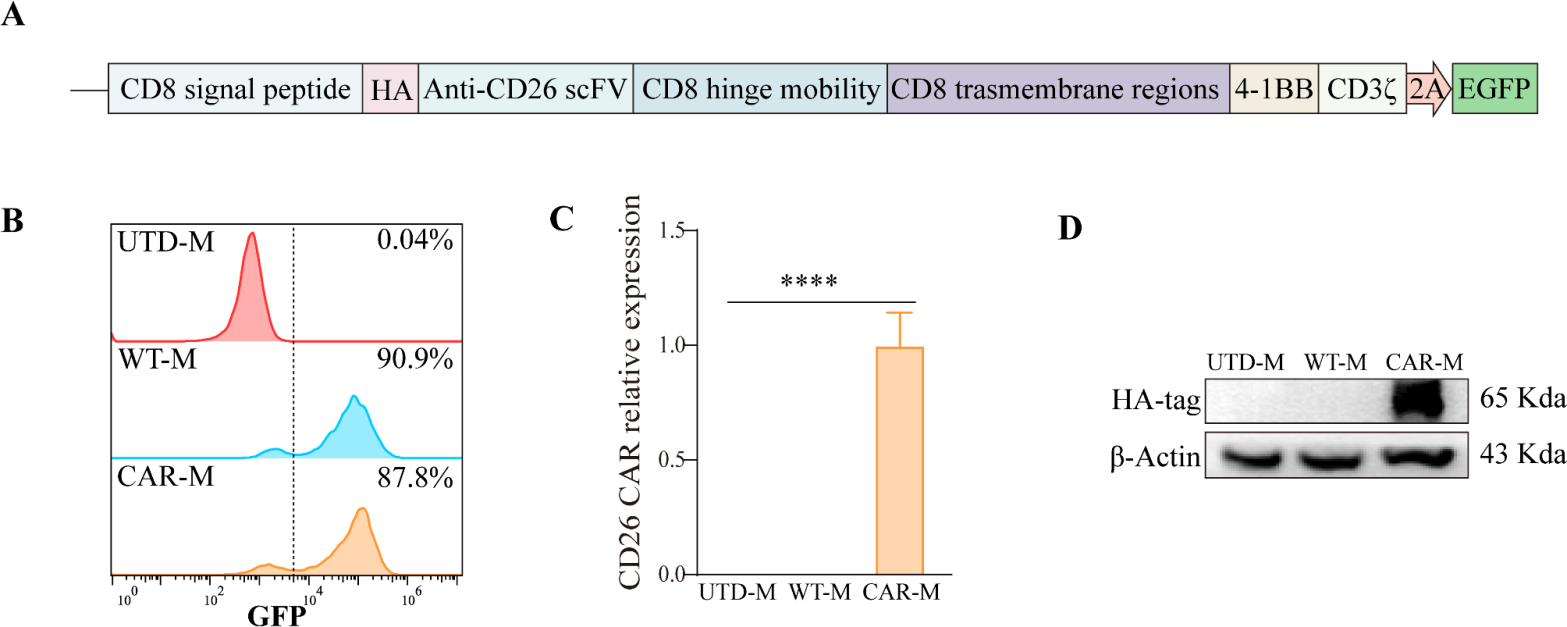
Generation and characterization of CD26 CAR-M. (**A**) Schematic of the CD26 CAR structure. The CAR comprises a signal peptide, a scFv recognizing CD26, a hinge mobility, a transmembrane region, and the costimulatory domains. Then, the CAR transgenes were linked to the EGFP-expressing sequence via a 2 A sequence to facilitate in vitro visualization. (**B**) The transfection efficiency was confirmed by detecting the fluorescence intensity of GFP by FCM. UTD-M are Raw264.7 cells without gene editing, and WT-M are Raw264.7 cells transfected by negative control lentivirus. (**C**) The CD26 CAR expression level in CAR-M was detected by qRT-PCR. (**D**) The expression of CAR was detected by measuring the protein expression level of HA-tag by Western blot. Experiments were repeated for three times, p-values was calculated with one-way ANOVA, and data represent the mean ± SD, **** *p* < 0.0001

We transduced RAW264.7 cells with a second-generation anti-CD26 CAR construct to generate CD26 CAR-M. The CARs comprise a CD8 signal peptide, a scFv recognizing human CD26, a CD8 hinge mobility and CD8 transmembrane region, and the intracellular domains including 4-1BB and CD3ζ. The CAR transgenes were linked to the EGFP-expressing sequence via a 2 A element. (Fig. 1A, Fig. S2A). The hinge region facilitates the scFv to target antigens. The transmembrane domain links the extracellular and intracellular domains of the CAR structure, facilitating the transmission of the ligand recognition signal into the cell. The co-stimulatory domain is responsible for transmitting the activation signal to the cells. Following lentiviral transduction, CAR expression in CD26 CAR-M cells reached 87.8% (Fig. 1B, Fig. S2B). qRT-PCR analysis demonstrated a significant upregulation of anti-CD26 scFv expression in the CAR-M cells, while Western blot analysis verified the presence of the CAR structure through the detection of HA-Tag expression (Fig. 1C-D). Collectively, these results indicate that the CD26 CAR was effectively expressed in CAR-M cells.

### Construction and identification of CD26-overexpressed CML cells

According to reports, CD26 expression is predominantly restricted to CML-LSCs and is absent in leukemia stem cells (LSCs) from other myeloid or lymphoid neoplasms [9]. Additionally, CD26 is not typically expressed on normal bone marrow stem cells, making it a novel and specific target for CML-LSCs [22]. To facilitate the experiment, we overexpressed CD26 in murine CML cells BP210 and BP210-T315I. The overexpression of CD26 was confirmed using FCM and qRT-PCR. Results demonstrated successful CD26 overexpression in both BP210 and BP210-T315I cells (Fig. S3A-C).

### CAR-M targeting phagocytosis in vitro

**Fig. 2:**
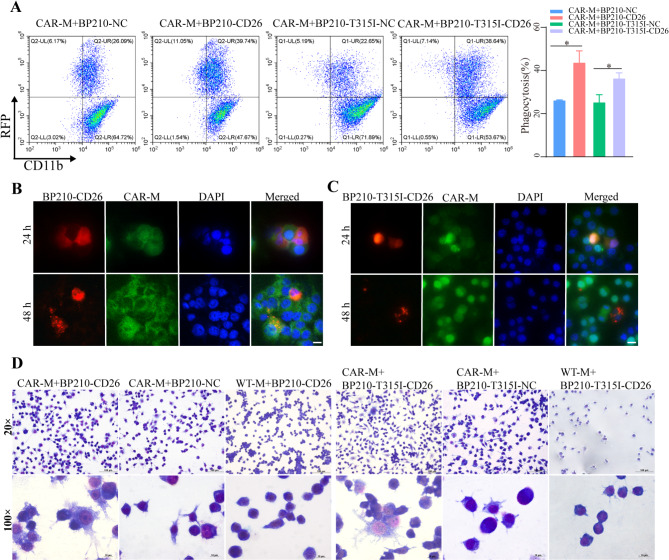
CAR-M targeting phagocytosis of CD26-positive CML cells in vitro. (**A**) The phagocytosis of CAR-M (CD11b+) on CD26-positive CML (RFP+) cells was detected by FCM as the percentage of double-positive cells (CD11b + and RFP+). (**B**-**C**) The CAR-M (green fluorescence) phagocytosis against CD26-positive CML cells (red fluorescence) was measured after 24–48 h of co-culture by CLSM, scale bar = 10 μm (**D**) Wright’s staining of co-cultured CAR-M and CML cells, scale bar = 100 μm for 20×, scale bar = 10 μm for 100×. Experiments were repeated for three times, p-values was calculated with one-way ANOVA, data were shown as mean ± SD, *p < 0.05

To assess CAR-mediated tumor phagocytosis, BP210-CD26 and BP210-T315I-CD26 cells were incubated with CAR-M or WT-M at the effect-target ratio of 5:1. The phagocytic effect of CAR-M on target cells was evaluated by flow cytometry. The results indicated that CAR-M had a higher phagocytic efficiency on CD26-positive cells compared to CD26-negative cells (Fig. 2A). Compared to WT-M cells, CAR-M has higher phagocytosis efficiency on CD26-positive cells (Fig. S4A). Macrophages have an inherent anti-leukemia activity [23], which may induce the background phagocytosis of CD26-negative cells. Nevertheless, the CD26 CAR increased the anti-leukemia activity of macrophages against CD26-positive cells. In addition, we used a fluorescent microscope to detect CAR-M phagocytosis in target cells. After 24 h of co-incubation, CAR-M showed evident fluorescence co-localization with the target cells, and after 48 h, the red fluorescence fragments of CD26-positive cells were surrounded by CAR-M’s green fluorescence, demonstrating that CAR-M could efficiently phagocytize CD26-positive cells (Fig. 2B-C). In addition, the CAR-M showed a higher phagocytosis on CD26-positive cells than WT-M (Fig. S4B-C). Wright’s staining indicated that CAR-M polarization and phagocytosis increased after co-culture with CD26-positive cells (Fig. 2D).

### Construction and phagocytic effect of CAR-THP1

**Fig. 3:**
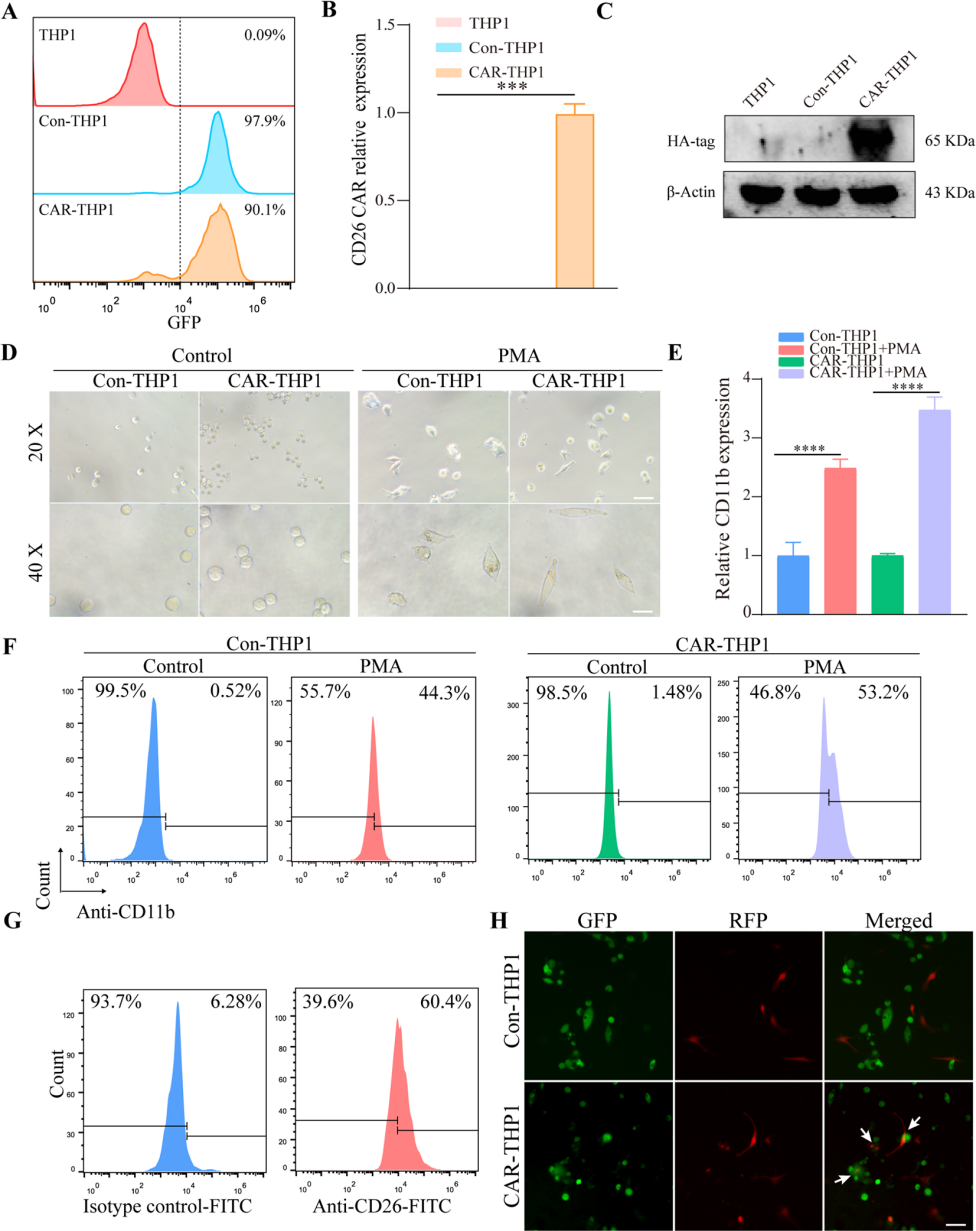
CD26 CAR-THP1 construction and phagocytic activity on CD26-positive cells. (**A**) The transfection efficiency was assessed by measuring the fluorescence intensity of GFP using FCM. (**B**) The mRNA expression level of CD26 CAR was detected by qRT-PCR. (**C**) The expression of CAR was detected by measuring the protein expression level of HA-tag by Western blot. (**D**) The morphology of CAR-THP1 and Con-THP1 was detected by macroscope after PMA (100 ng/ml, 48 h) treatment, scale bar = 20 μm for 20×, scale bar = 10 μm for 40×. (**E**) The expression level of macrophage marker (CD11b) in CAR-THP1 and Con-THP1 after PMA (100 ng/ml, 48 h) treatment was detected by qRT-PCR. (**F**) The expression of macrophage marker (CD11b) on CAR-THP1 and Con-THP1 after PMA (100 ng/ml, 48 h) treatment was measured by FCM. (**G**) The expression of CD26 on NCI-H2452 was measured by FCM. (**H**) The phagocytic effect of CAR-THP1 and Con-THP1 (green fluorescence) on NCI-H2452 (red fluorescence) was detected by CLSM, scale bar = 20 μm. Experiments were repeated for three times, statistical significance was calculated using one-way ANOVA, data were shown as mean ± SD, ***p < 0.001, ****p < 0.0001

THP1 is a human myeloid leukemia monocytic cell line that can be induced for differentiation into macrophages using PMA. The CD26 CAR-THP1 was constructed, and the efficiency of lentiviral transfection was confirmed by flow cytometry and fluorescence analysis (Fig. 3A, Fig. S5A). Results of qRT-PCR and Western blot indicated that the CD26 CAR was significantly expressed in CAR-THP1 cells (Fig. 3B-C). Subsequently, PMA was used to induce the differentiation of CD26 CAR-THP1 into macrophages. The differentiation efficacy was verified by flow cytometry, qRT-PCR, and microscopy. THP1 cells displayed increased adhesion, transitioned from a suspended to an adherent state, exhibited morphological changes from spherical to irregular shapes, and increased in volume (Fig. 3D) Moreover, qRT-PCR and FCM analyses revealed an upregulation of CD11b expression in THP1 cells following PMA treatment (Fig. 3E-F). NCI-H2452, a malignant mesothelioma cell line that naturally overexpresses CD26, was transduced with lentivirus to overexpress RFP (Fig. 3G, Fig. S5B). To verify the specifically targeting capacity of CAR-THP1, NCI-H2452 cells were co-cultured with CAR-THP1 or Con-THP1. Fluorescence microscopy results indicated that CAR-THP1 demonstrated increased phagocytosis of NCI-H2452 cells than Con-THP1 (Fig. 3H).

### Safety of CAR-M cells

**Fig. 4:**
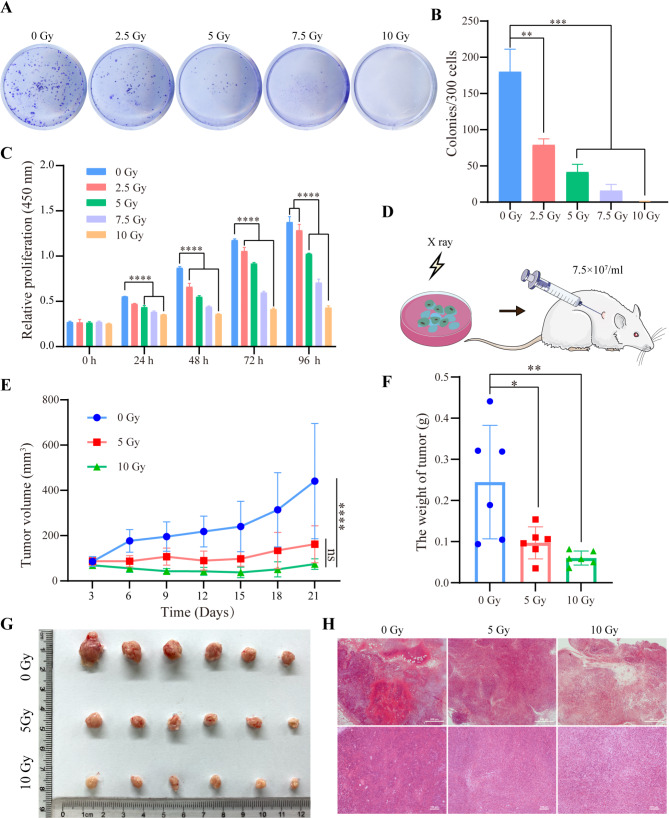
The safety of CAR-M was evaluated both in vitro and in vivo. (**A**-**B**) The clone-forming ability of CAR-M was detected after X-ray irradiation. (**C**) The proliferation capability of CAR-M after X-ray irradiation was detected by CCK-8. (**D**) The experimental flow to evaluate the safety of CAR-M in vivo. BALB/c mice (5–6 weeks old) received subcutaneous injections of 1 × 10⁷ untreated, 5 Gy-irradiated, or 10 Gy-irradiated CAR-M cells. (**E**) The tumor volume, (**F**) the tumor weight, and (**G**) the tumor image of mice after 21 days of CAR-M injection. (**H**) The infiltration of CAR-M in tumor tissue was detected by HE staining, scale bars: 500 μm and 100 μm. P-values are calculated using one‐way ANOVA, data are presented as mean ± SD (*n* = 3 in vitro, *n* = 6 in vivo), ns: not significant, *p < 0.05, **p < 0.01, ***p < 0.001, ****p < 0.0001

Given that CAR-M cells were derived from the immortalized Raw264.7 cell line, X-ray irradiation was necessary before in vivo application to mitigate tumorigenicity. To determine the optimal irradiation dose for safety, CAR-M cells were exposed to X-rays at doses of 0, 2.5, 5, 7.5, and 10 Gy. Proliferation capacity was evaluated using CCK-8 and colony formation assays. The colony formation assay indicated a marked reduction in colony numbers following irradiation at doses between 5 and 10 Gy after 7 days (Fig. 4A-B). CCK-8 analysis further confirmed that the proliferation of CAR-M cells was significantly inhibited post-irradiation (Fig. 4C). To assess the in vivo tumorigenic potential, CAR-M cells were irradiated and injected into mice (Fig. 4D). As a result, the tumor size and weight were significantly lower in the 5 Gy and 10 Gy irradiation groups compared to the non-irradiated control group (Fig. 4E-G). HE staining revealed a substantial reduction in tumor cell infiltration in the 5 Gy and 10 Gy groups, with the 10 Gy group showing the lowest level of infiltration (Fig. 4H). These results demonstrated that 10 Gy X-ray irradiation significantly reduces the tumorigenicity of CAR-M cells. Therefore, CAR-M cells will be pre-treated with 10 Gy X-ray irradiation for all subsequent in vivo experiments.

### Anti-leukemia capability of CAR-M in vivo

**Fig. 5:**
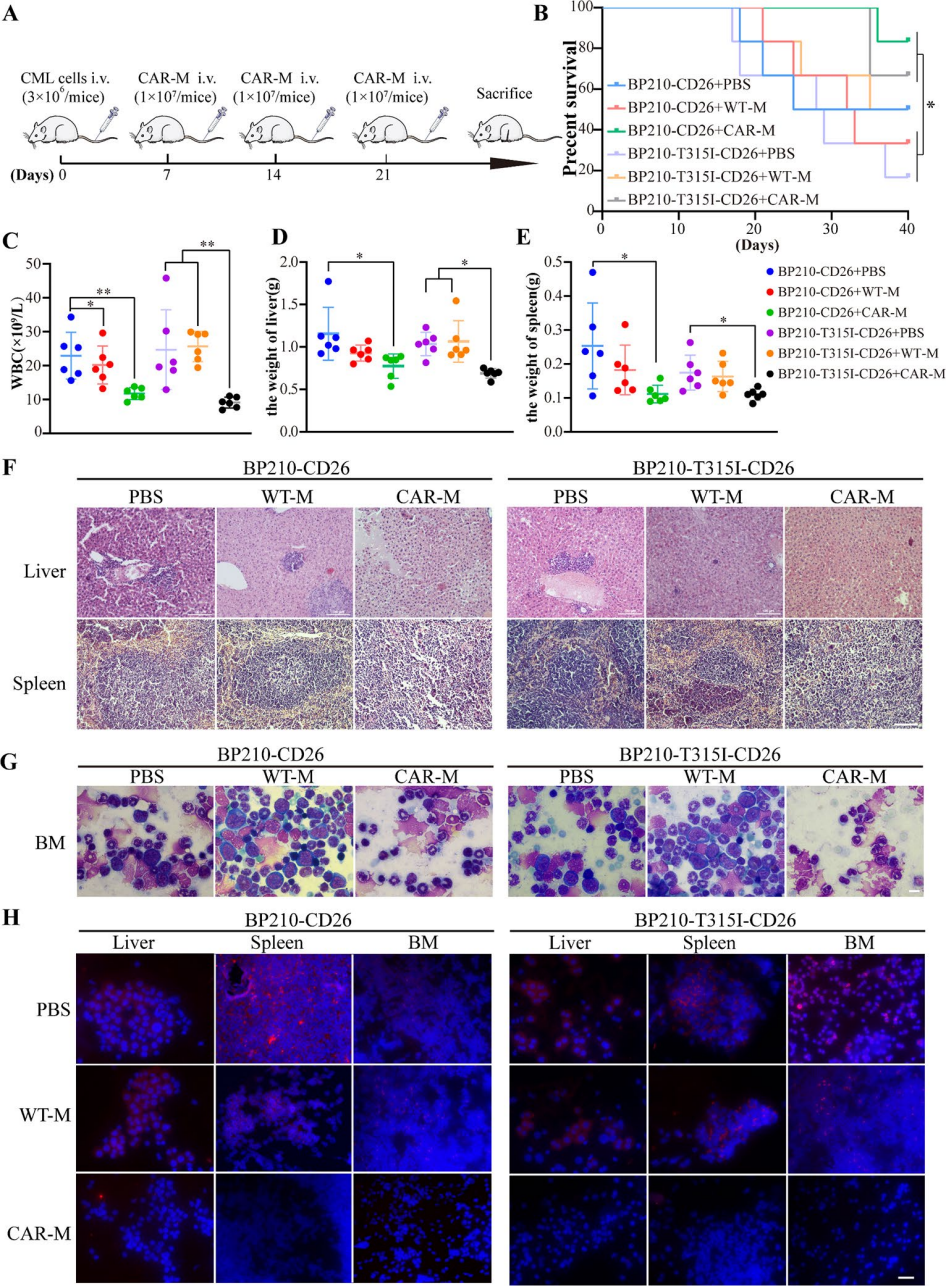
The anti-leukemia capability of CAR-M was assessed in vivo. (**A**) The experimental flow to evaluate the anti-leukemia effects of CAR-M in vivo. Mice were intravenously injected with 1 × 10^7^ CD26 + BP210 or CD26 + BP210-T315I on day 0. Then, 1 × 10^7^ irradiated CAR-M or WT-M cells were injected on days 7, 14, and 21, while the blank group received PBS. (**B**) Survival curves were analyzed by Kaplan–Meier methods using GraphPad Prism 8.0. (**C**) The peak WBC count, (**D**) liver weight, and (**E**) spleen weight of mice was recorded. (**F**) HE staining was used to detect the CML cell infiltration in the liver and spleen of mice, scale bar = 100 μm. (**F**) Morphology of the BM was detected by Wright’s stain, scale bar = 10 μm. (**H**) The expression of BCR/ABL in the liver, spleen, and BM of mice was detected by immunofluorescence, scale bar = 50 μm. P-values are calculated using one‐way ANOVA, data were shown as mean ± SD (*n* = 6 in each group), *p < 0.05, **p < 0.01

Female BALB/c mice (5–6 weeks old) were randomly divided into 6 groups (*n* = 6). The mice were injected with 3 × 10^6^ CD26^+^BP210 or CD26^+^BP210-T315I cells via the tail vein (day 0). One week later, the experimental group mice were injected with 1 × 10^7^ CAR-M cells irradiated with 10 Gy X-ray through the tail vein, the control group was injected with an equal count of WT-M cells, and the blank group was injected with PBS (Fig. 5A). The injections were repeated on days 14 and 21. Kaplan-Meier survival analysis demonstrated that CAR-M-treated mice exhibited significantly longer survival times compared to those in the other groups (Fig. 5B). The mice’s body weight and WBC count were measured weekly. Figure 5C shows that CAR-M-treated mice had significantly lower peak WBC levels compared to other groups. Additionally, the mice in the PBS and WT-M groups showed more severe hepatosplenomegaly compared to the CAR-M group (Fig. 5D-E, Fig. S6A). Histological examination through HE and Wright’s staining showed that the CAR-M treatment group experienced significantly less leukemic cell infiltration in the liver and spleen compared to the PBS and WT groups (Fig. 5F, Fig. S6B). Wright’s staining of the BM revealed that the CAR-M group had less infiltration of leukemic cells than the other groups (Fig. 5G). Immunofluorescence analyses further demonstrated decreased expression of the BCR::ABL1 protein in the liver, spleen, and BM of the CAR-M-treated group relative to the other groups (Fig. 5H). In conclusion, these results confirm that CAR-M therapy effectively reduces the oncogenic potential of CML cells, including those harboring the T315I mutation, in vivo.

**Fig. 6:**
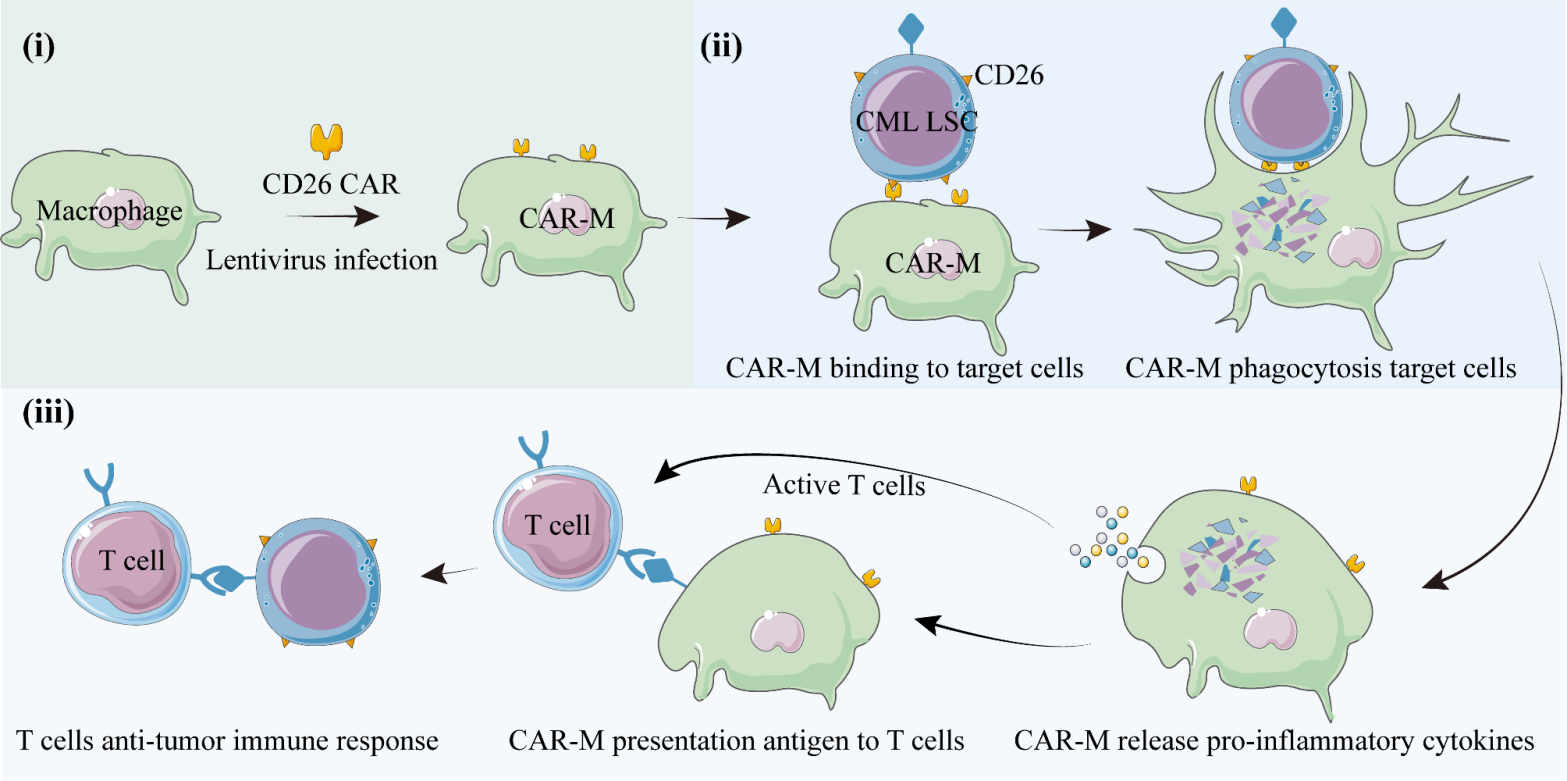
The diagram of CAR-M targeting CD26 to eliminate chronic myeloid leukemia stem cells. (**i**) The CD26 CAR-M was constructed by lentivirus infection. (**ii**) CD26 CAR-M target and engulf CD26-positive CML-LSCs. (**iii**) CAR-M releases pro-inflammatory cytokines and active adaptive anti-tumor immunity

### Immune activation effect of CAR-M

Macrophages, as one of the main antigen-presenting cells, can not only phagocytose target cells but also present target cell antigens, thereby stimulating endogenous anti-tumor T cell responses and activating the anti-tumor immune system [24]. To assess the immune activation effect of CAR-M cells, we evaluated cytokine expression after co-culturing CAR-M and target cells. The qRT-PCR results revealed a significant increase in the expression levels of IL-1β, IL-6, and TNF-α (Fig. S7).

## Discussion

CML is a malignant proliferative disease derived from BM hematopoietic stem cells, and TKIs function by inhibiting the tyrosine kinase activity of the pathogenic protein BCR::ABL1 in CML cells. However, CML-LSCs exhibit intrinsic resistance to TKIs therapy and serve as the source of TKIs resistance, relapse, and disease progression, forcing the urgent need for novel therapeutic strategies [25]. Currently, there are various strategies for targeting CML-LSCs. For instance, drug combination therapies selectively inhibit plasminogen activator inhibitor-1 (PAI-1) to enhance the susceptibility of CML-LSCs to TKIs [30]. Targeting pyruvate carboxylase-mediated anaplerosis is a promising therapeutic approach for CML-LSCs treatment [31]. Furthermore, research has revealed that reprogramming of enhancer of zeste homolog 2 (EZH2) and H3K27me3 is crucial for CML-LSCs survival, suggesting a combined treatment approach with EZH2i and TKIs could improve therapeutic efficacy [32]. CAR therapy, as a novel form of immunotherapy, has shown groundbreaking outcomes in the treatment of hematological malignancies.

Several studies have investigated the possibility of CAR therapies for CML treatment. CD38 CAR-T may overcome TKIs and chemotherapy resistance in patients with myeloid blast phase CML (CML-BP). However, as CD38 is expressed in normal stem cells, targeting CD38 has serious side effects, so CD38 CAR may be an option for therapy for myeloid CML-BP patients who could be eligible for BM transplant [26]. CD25, IL-1RAP, and CD26 were found to be highly expressed in CML-LSC**s** [10]. IL-1RAP CAR-T therapy exhibited cytotoxicity against CML-LSCs, but it also killed normal monocytes [27]. CD25 CAR-NK demonstrated excellent anti-leukemic activity against BP-CML, indicating a novel potential therapy strategy [28]. However, the amount of IL-1RAP and CD25 will downregulate in CML-LSCs after TKIs treatment, influencing the targeting efficacy of CAR therapy [29, 30]. CD26 has been identified as a specific marker of CML-LSCs, highly expressed in CML-LSCs and extremely low in normal stem cells. The expression of CD26 was maintained in CML-LSCs during TKIs treatment and after TKIs discontinuation [31]. Therefore, targeting CD26 might be more effective for eliminating CML-LSCs.

Although the Food and Drug Administration (FDA) approved CAR-T cell therapy in multiple cancer treatments, CRS, immune effector cell-associated neurotoxicity syndrome (ICANS), and hemocytopenia pose hurdles in the use of CAR-T therapy in patients [32]. Moreover, the exhaustion of CAR-T cells is a critical factor contributing to treatment failure and disease recurrence, often linked to functional deficiencies mediated by c-Jun in exhausted T cells [33]. CAR-NK cells are safer than CAR-T cell therapy since they release neither IL-1 nor IL-6 and seldom induce graft-versus-host disease (GVHD) [16]. However, limitations such as the inability of NK cells to survive long-term in vivo and their inherent resistance to lentivirus-based transduction restrict the therapeutic potential of CAR-NK cells [34]. CAR-M showed significant potential in CAR-related therapies. The recent advances in CAR-M technology have shown its ability to penetrate tumor microenvironments effectively, enhancing the treatment of solid tumors. CAR-M not only engulfs tumor cells but also has an antigen presentation function, boosting T cell activity. Moreover, CAR-M has a short extracorporeal circulation time and low non-tumor-targeted toxicity. Research has demonstrated that GD2-CAR-M is effective in killing neuroblastoma and melanoma cells that express GD2 [35]. CD147 CAR-M had improved anti-tumor efficacy against K562 and MDA-MB-231 cells, with minimal off-target cytotoxicity against normal cells [36]. CT-0508, a human epidermal growth factor receptor 2 (HER2)-targeted CAR-M, has received fast-track designation by the FDA for the treatment of solid tumors [18]. MCY-M11, a CAR-M therapy targeting mesothelioma, has been approved by the FDA for clinical trials [17]. Those making CAR-M therapies a significant step towards practical therapeutic applications. Despite these advances, the application of CAR-M in hematological malignancies remains underexplored, necessitating further research.

CAR-M therapy is a promising tumor treatment strategy with distinctive phagocyitc capabilities, antigen-presenting prorerties, and the ability to activate adaptive immunity (Fig. 6).In this study, we constructed CD26 CAR-M using mouse-derived macrophages Raw264.7 and overexpressed CD26 in murine CML cell lines, BP210 and BP210-T315I. The results indicate that CAR-M effectively mediates targeted phagocytosis of CD26-positive CML cells both in vitro and in vivo (Fig. 2). Furthermore, we developed CAR-THP1 and evaluated its phagocytic capabilities on NCI-H2452 cells that naturally express CD26. The data demonstrate that CAR-M exhibited significantly enhanced phagocytic efficacy against CD26-positive cells compared to CD26-negative cells, suggesting that CD26 CAR-M cell therapy may represent a promising treatment modality for CML.

The CAR configurations influence phagocytosis and targeting efficiency. The structure of CAR-M is similar to CAR-T cells, but there are significant differences in their intracellular signaling, adding special co-stimulatory molecules helps enhance the anti-tumor effect of CAR-M. In addition to CD3ζ, adding the Fc receptor g subunit (FcRg) [37], multiple epidermal growth factor-like domain protein 10 (Megf10) [38], and tyrosine-protein kinase Mer (MerTK) [39] can promote CAR-M phagocytosis. Macrophage cells have M1 and M2 types. M1 macrophages exhibit anti-tumor activity through phagocytosis, tumor antigen presentation, and pro-inflammatory factors. To the contrary, M2 macrophages have pro-tumor activity [40]. Therefore, redirecting macrophages towards M1 may enhance CAR-M activity. A second-generation CAR-M has been developed by integrating intracellular CD3ζ and toll-like receptor 4 intracellular toll/IL-1R (TIR) domains, with the capability to drive M1-like macrophage polarization, exhibiting superior anti-tumor functions relative to first-generation CAR-M [41].

There are limitations in this study as well. First, the use of mouse-derived macrophages and CML cell lines for experimental purposes does not fully replicate the complex tumor microenvironment found in humans. Future investigations will involve the utilization of macrophages derived from induced pluripotent stem cells (iPSCs) and the isolation of CD34^+^ stem cells from CML patients to validate the targeted phagocytic effects of CAR-M. Furthermore, while CD26 is prominently expressed in CML-LSCs, it is also present in certain normal cells, raising concerns regarding the potential cytotoxic effects of CD26 CAR-M on healthy tissues. As CD26 is expressed on normal T cells, CD26 CAR-M cell therapy is expected to cause lymphopenia as a side effect, necessitating future clinical trials to monitor T cell levels [42]. Furthermore, CD26 expression was observed in vascular endothelia, fibroblasts, and epithelium cells of colon, side effect may be caused by CD26 CAR-M treatment [43]. Previous studies have identified antigens, such as CD25 [44], CD93 [45], and IL1RAP [30] which are also highly expressed in CML-LSCs. Therefore, the development of a dual-target CAR-M strategy may enhance the specificity of targeting CML-LSCs while minimizing off-target effects. Considerations of CAR-M cell limitations should be mentioned as well. Similar to CAR-T, CAR-M has the risk of causing off-target toxicity and CRS. Regulating CAR-M polarization to M1 type is crucial, as M1 macrophages have anti-tumor qualities while M2 macrophages are pro-tumor. Additionally, a significant proportion of CAR-M cells reside in the liver, potentially impairing their circulation and tumor-targeting capabilities. The impact of this phenomenon on liver function warrants thorough investigation in future CAR-M research [46].

The clinical translation of CAR-M requires attention to several aspects. First of all, the safety and efficacy of CAR-M treatment must be proved in human trials. The CAR components have non-patient-derived sequences and may have inherent immunogenicity. Using humanized scFv and adding a suicide gene helps improve the safety of CAR-M cells [47]. CAR-M therapy needs frequent administration of a large number of CAR-M cells. However, there are limited human macrophages in peripheral blood, and they cannot proliferate in vitro. Inducing iPSCs to CAR-M could contribute to the scalability of CAR-M production [48]. Preparing CAR-M through viral transfection may induce insertion mutations. Using biocompatible materials, such as nanoparticles, to deliver gene editing agents may provide opportunities for the safe production of CAR-M [49].

## Conclusion

This study successfully constructed CD26 CAR-M, which effectively exhibited specific phagocytosis of CD26-positive CML cells both in vitro and in vivo. This approach highlights the potential to eliminate CML-LSCs, offering promising therapeutic options for patients with relapsed or resistant CML following TKIs treatment. Additionally, our findings provide valuable insights into the application of CAR-M in the treatment of hematological malignancies.

## Electronic supplementary material

Below is the link to the electronic supplementary material.

Supplementary Material 1: Supplementary Fig. 1. The construction of CAR-T and CAR-NK. (A) The expression of CD26 was detected by FCM, UTD-T is T cells without gene editing, WT-T is T cells with GFP expression, and CD26 CAR-T is T cells infected by CD26 CAR lentivirus. (B) The proliferation of CD26 CAR-T was detected by CCK-8. (C) The apoptosis of CD26 CAR-T was detected by FCM. (D) The GFP fluorescence expression in NK-92 and KCL22 cells after 72 h of CD26 CAR lentivirus transfection, scale bar = 50 μm. Experiments were repeated for three times, statistical significance was calculated using one-way ANOVA, data were shown as mean ± SD, ns: not significant, **p < 0.01, ***p < 0.001, ****p < 0.0001

Supplementary Material 2: Supplementary Fig. 2. Characteristics of CAR-M after lentivirus infection. (A) The viral vector structure of CD26 CAR. (B) The fluorescent microscopic images of WT-M and CAR-M after 72 h of CD26 CAR lentivirus transfection, scale bar = 100 μm

Supplementary Material 3: Supplementary Fig. 3. The construction of a CD26-positive CML cell line. (A) The fluorescent microscopic images of CML cells, scale bar = 100 μm. (B) The transfection efficiency was confirmed by detecting the fluorescence intensity of RFP by FCM. (C) The mRNA level of CD26 in CML cells was detected by qRT-PCR. P-values are calculated using one‐way ANOVA, data were shown as mean ± SD (*n* = 3), ****p < 0.0001

Supplementary Material 4: Supplementary Fig. 4. The phagocytosis capability of CAR-M and WT-M on CD26-positive CML cells. (A) The phagocytosis of CAR-M and WT-M on CD26-positive CML cells was detected by FCM. (B) The phagocytosis of CAR-M and WT-M on CD26-positive CML cells (red fluorescence), the white arrow indicates the phagocytosis of cells, scale bar = 20 μm (C) Quantitative analysis of data from (B). The phagocytic cells in three random fields were assessed. P-values are calculated using one‐way ANOVA, data were shown as mean ± SD (*n* = 3), *p < 0.05

Supplementary Material 5: Supplementary Fig. 5. The fluorescent label for CAR-THP1 and NCI-H2452. (A) The fluorescent microscopic images of Con-THP1 and CAR-THP1, scale bar = 100 μm. (B) The fluorescent microscopic images of NCI-H2452, scale bar = 50 μm

Supplementary Material 6: Supplementary Fig. 6. Characteristics of the liver and spleen of CML mice. (A) The image of the liver and spleen in CML mice. (B) The infiltration of CML cells in the liver and spleen was detected by Wright’s stain, scale bar = 10 μm

Supplementary Material 7: Supplementary Fig. 7. Immune activation effect of CAR-M. The relative expression level of TNF-α, IL-1β, and IL-6 in co-culture system. Experiments were repeated for three times, p-values are calculated using one‐way ANOVA, results are mean ± SD, **p < 0.01, ****p < 0.0001

Supplementary Material 8: Supplementary Table. Primer sequence list

## Data Availability

No datasets were generated or analysed during the current study.

## Abbreviations

CML-LSCs: Chronic myeloid leukemia stem cells
BM: Bone marrow
CML: Chronic myeloid leukemia
TKIs: Tyrosine kinase inhibitors
LSCs: Leukemia stem cells
CAR: Chimeric antigen receptor
TSA: Tumor-specific antigen
scFv: Single-chain variable fragment
CAR-NK: CAR natural killer
CAR-M: CAR macrophages
FCM: Flow cytometry
CRS: Cytokine release syndrome
CML-CP: Chronic phase CML
qRT-PCR: Quantitative reverse transcription PCR
CLSM: Confocal laser scanning microscopy
RFP: Red fluorescent protein
WBC: White blood cell
HE: Hematoxylin and eosin
CML-BP: Blast phase CML
FDA: Food and Drug Administration
iPSCs: Pluripotent stem cells
