# Supplementary figures and images for: CAR-macrophages targets CD26 to eliminate chronic myeloid leukemia stem cells
Source: Exp Hematol Oncol. 2025 Feb 13;14:14. doi: 10.1186/s40164-025-00608-9 (PMC11823019; doi:10.1186/s40164-025-00608-9)

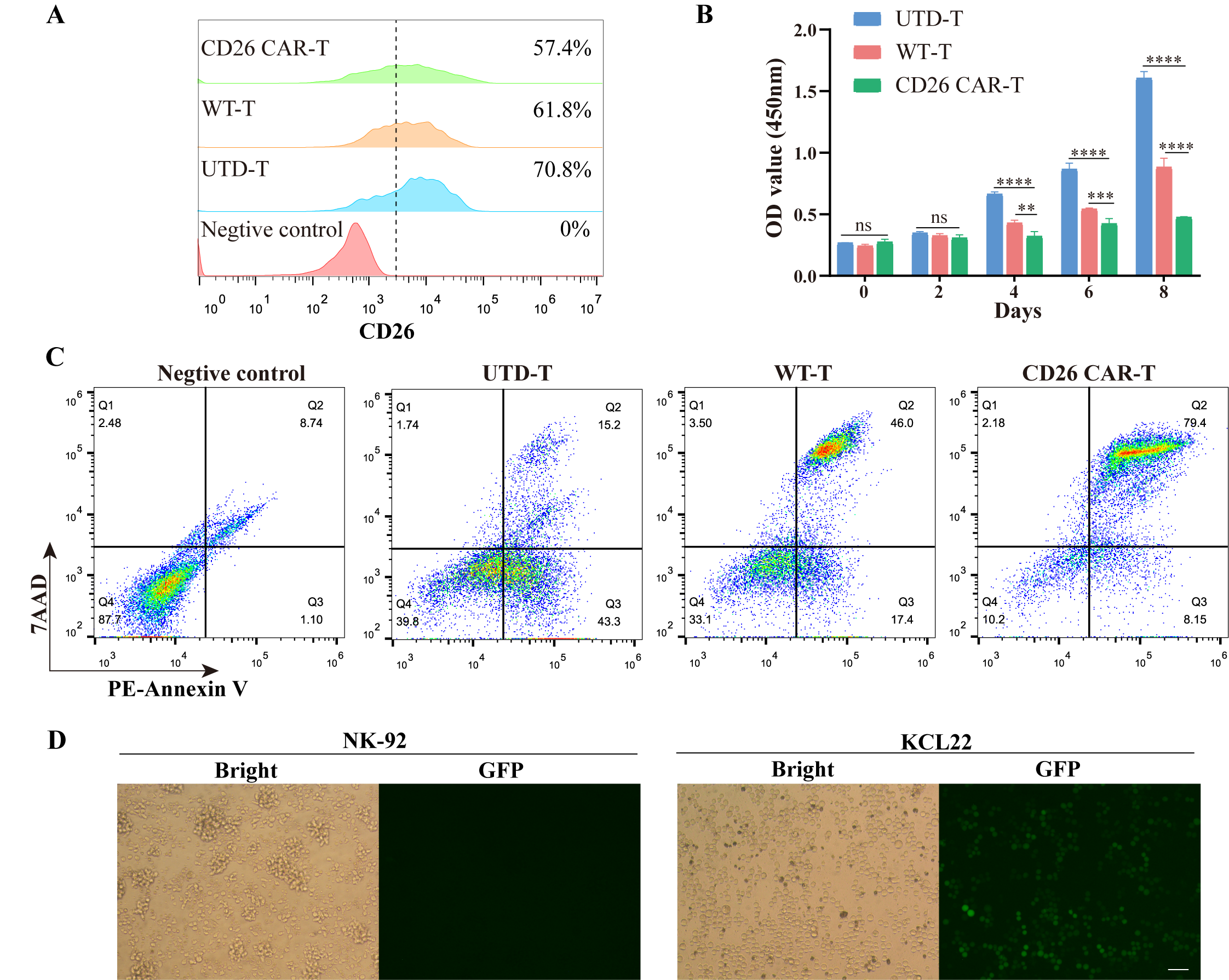

Supplement: Supplementary file 1 — Supplementary Material 1: Supplementary Fig. 1. The construction of CAR-T and CAR-NK. (A) The expression of CD26 was detected by FCM, UTD-T is T cells without gene editing, WT-T is T cells with GFP expression, and CD26 CAR-T is T cells infected by CD26 CAR lentivirus. (B) The proliferation of CD26 CAR-T was detected by CCK-8. (C) The apoptosis of CD26 CAR-T was detected by FCM. (D) The GFP fluorescence expression in NK-92 and KCL22 cells after 72 h of CD26 CAR lentivirus transfection, scale bar = 50 μm. Experiments were repeated for three times, statistical significance was calculated using one-way ANOVA, data were shown as mean ± SD, ns: not significant, **p < 0.01, ***p < 0.001, ****p < 0.0001 [file 40164_2025_608_MOESM1_ESM.tif]

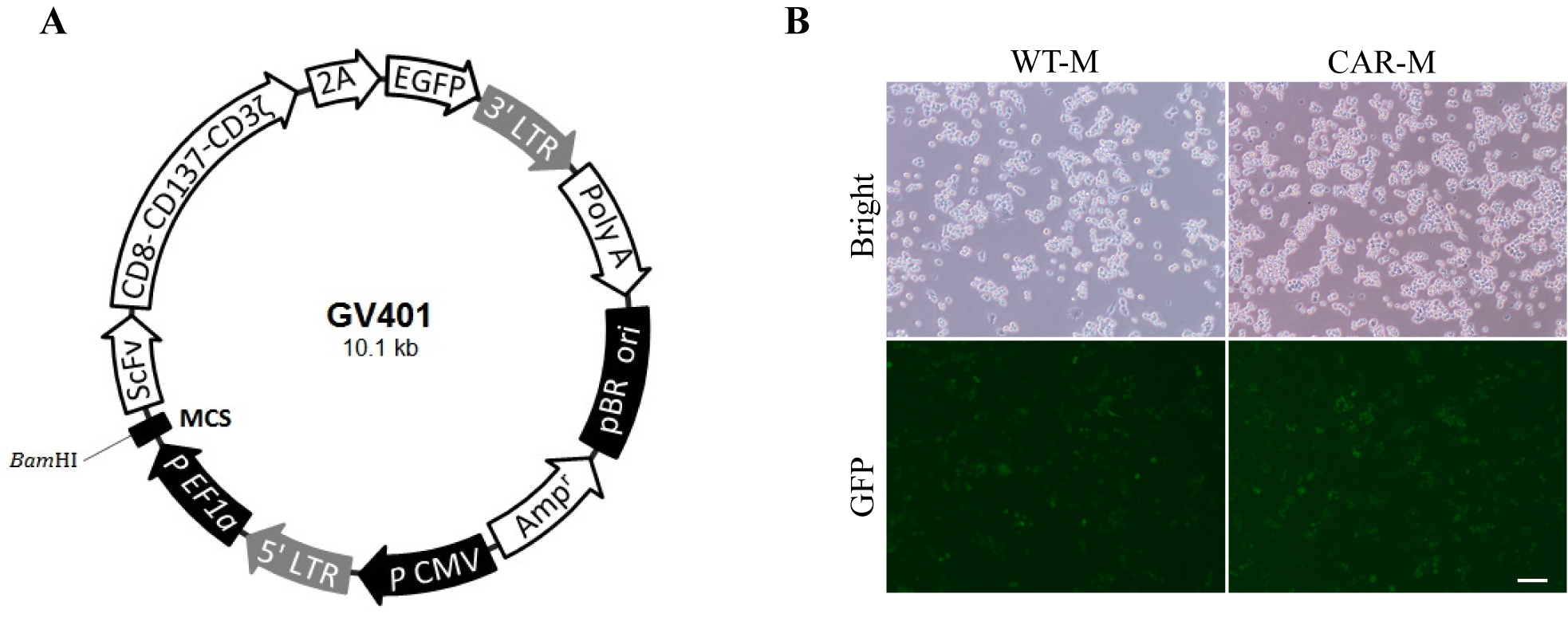

Supplement: Supplementary file 2 — Supplementary Material 2: Supplementary Fig. 2. Characteristics of CAR-M after lentivirus infection. (A) The viral vector structure of CD26 CAR. (B) The fluorescent microscopic images of WT-M and CAR-M after 72 h of CD26 CAR lentivirus transfection, scale bar = 100 μm [file 40164_2025_608_MOESM2_ESM.tif]

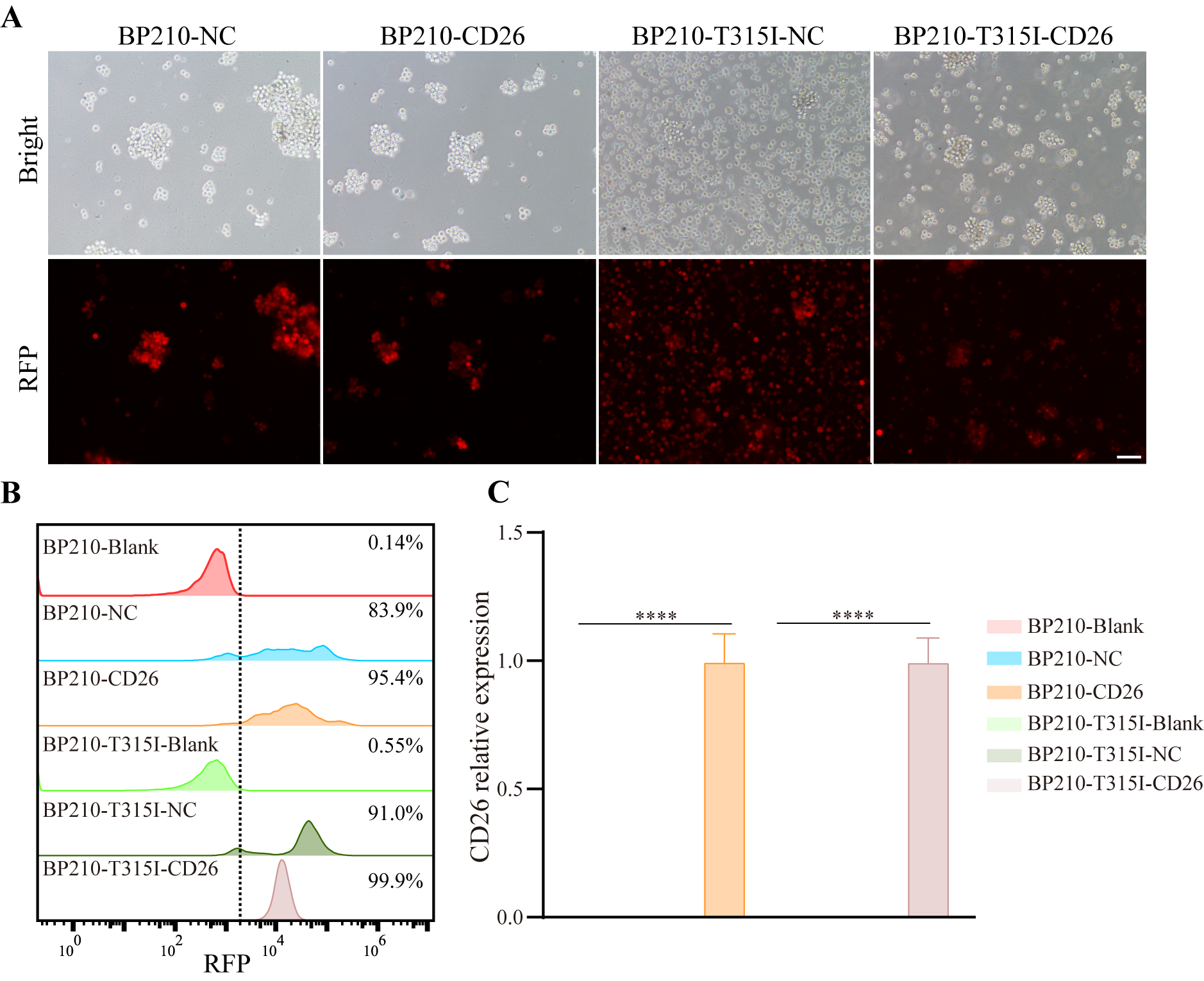

Supplement: Supplementary file 3 — Supplementary Material 3: Supplementary Fig. 3. The construction of a CD26-positive CML cell line. (A) The fluorescent microscopic images of CML cells, scale bar = 100 μm. (B) The transfection efficiency was confirmed by detecting the fluorescence intensity of RFP by FCM. (C) The mRNA level of CD26 in CML cells was detected by qRT-PCR. P-values are calculated using one‐way ANOVA, data were shown as mean ± SD (n = 3), ****p < 0.0001 [file 40164_2025_608_MOESM3_ESM.tif]

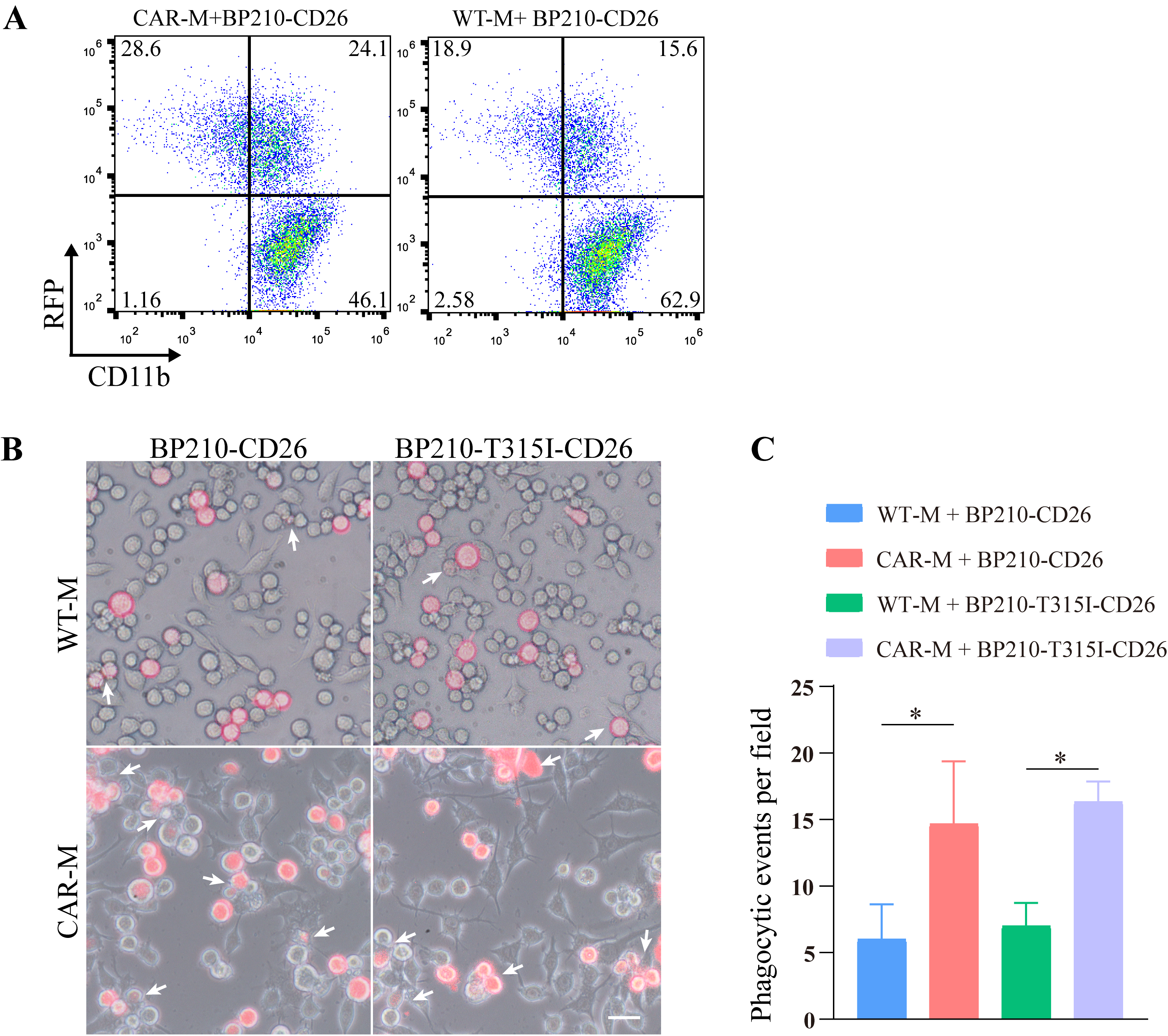

Supplement: Supplementary file 4 — Supplementary Material 4: Supplementary Fig. 4. The phagocytosis capability of CAR-M and WT-M on CD26-positive CML cells. (A) The phagocytosis of CAR-M and WT-M on CD26-positive CML cells was detected by FCM. (B) The phagocytosis of CAR-M and WT-M on CD26-positive CML cells (red fluorescence), the white arrow indicates the phagocytosis of cells, scale bar = 20 μm (C) Quantitative analysis of data from (B). The phagocytic cells in three random fields were assessed. P-values are calculated using one‐way ANOVA, data were shown as mean ± SD (n = 3), *p < 0.05 [file 40164_2025_608_MOESM4_ESM.tif]

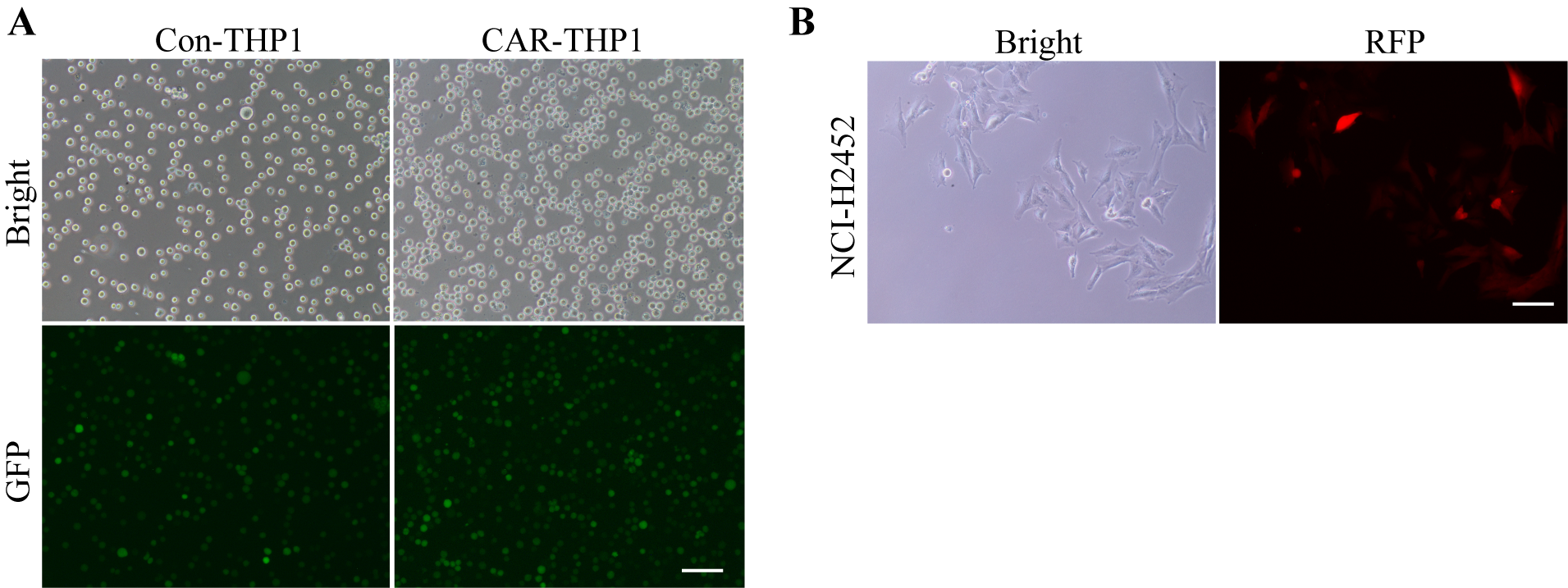

Supplement: Supplementary file 5 — Supplementary Material 5: Supplementary Fig. 5. The fluorescent label for CAR-THP1 and NCI-H2452. (A) The fluorescent microscopic images of Con-THP1 and CAR-THP1, scale bar = 100 μm. (B) The fluorescent microscopic images of NCI-H2452, scale bar = 50 μm [file 40164_2025_608_MOESM5_ESM.tif]

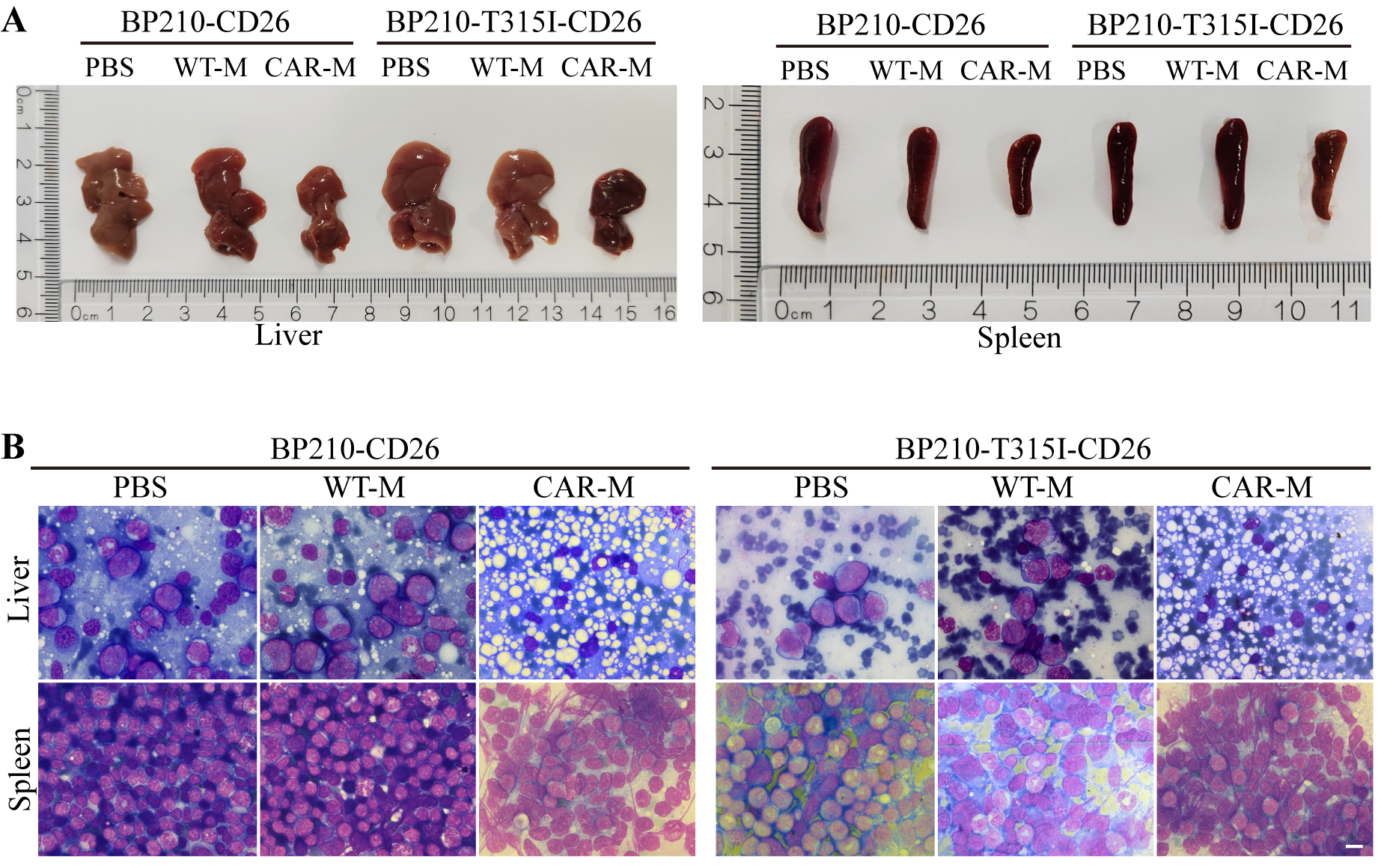

Supplement: Supplementary file 6 — Supplementary Material 6: Supplementary Fig. 6. Characteristics of the liver and spleen of CML mice. (A) The image of the liver and spleen in CML mice. (B) The infiltration of CML cells in the liver and spleen was detected by Wright’s stain, scale bar = 10 μm [file 40164_2025_608_MOESM6_ESM.tif]

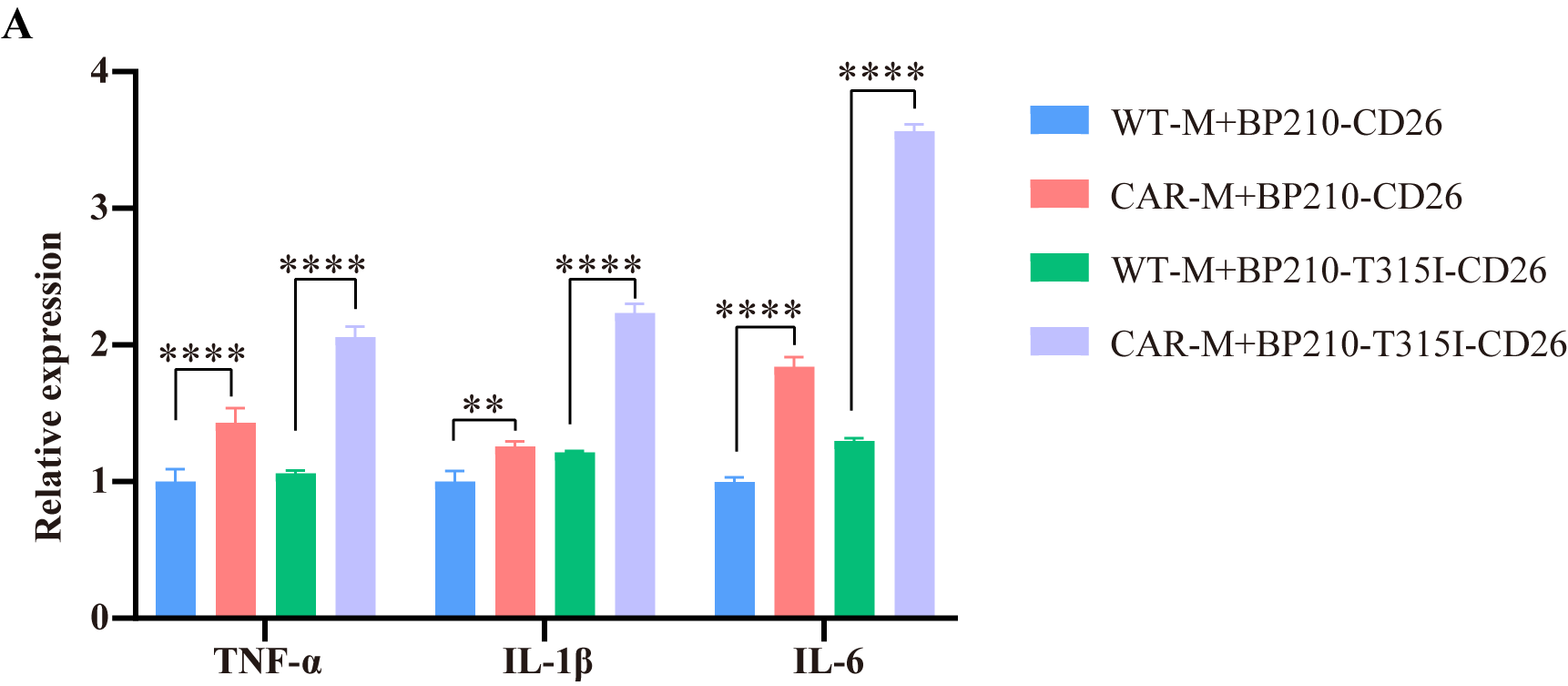

Supplement: Supplementary file 7 — Supplementary Material 7: Supplementary Fig. 7. Immune activation effect of CAR-M. The relative expression level of TNF-α, IL-1β, and IL-6 in co-culture system. Experiments were repeated for three times, p-values are calculated using one‐way ANOVA, results are mean ± SD, **p < 0.01, ****p < 0.0001 [file 40164_2025_608_MOESM7_ESM.tif]
