# Supplementary material for: CAR-macrophages targets CD26 to eliminate chronic myeloid leukemia stem cells
Source: Exp Hematol Oncol. 2025 Feb 13;14:14. doi: 10.1186/s40164-025-00608-9 (PMC11823019; doi:10.1186/s40164-025-00608-9)
Supplement: Supplementary file 8 — Supplementary Material 8: Supplementary Table. Primer sequence list [file 40164_2025_608_MOESM8_ESM.docx]

**Supplementary Table. Primer sequence list**

| **Gene** | **Forward** | **Reverse** |
| --- | --- | --- |
| Human β-Actin | GGCGGCACCACCATGTACCCT | AGGGGCCGGACTCGTCATACT |
| CD26 CAR | CCTTTAGCAGCTACGCCATG | GAGTTATCCCGTGAGATTGTGA |
| Human CD26 | AAAGGCACCTGGGAAGTCATCG | CAGCTCACAACTGAGGCATGTC |
| Human CD11b | GGAACGCCATTGTCTGCTTTCG | ATGCTGAGGTCATCCTGGCAGA |
| Mouse IL-1β | TGGACCTTCCAGGATGAGGACA | GTTCATCTCGGAGCCTGTAGTG |
| Mouse IL-6 | TACCACTTCACAAGTCGGAGGC | CTGCAAGTGCATCATCGTTGTTC |
| Mouse TNF-α | GGTGCCTATGTCTCAGCCTCTT | GCCATAGAACTGATGAGAGGGAG |
| Mouse β-Actin | CATTGCTGACAGGATGCAGAAGG | TGCTGGAAGGTGGACAGTGAGG |
